# The complete mitochondrial genome of *Anas zonorhyncha* (Swinhoe, 1866) (Anatidae: Anas)

**DOI:** 10.1080/23802359.2022.2064246

**Published:** 2022-04-24

**Authors:** Kuo Xu, Chuyu Lin, Xinyuan Zhang, Huabing Guo

**Affiliations:** a School of Biological Science, The University of Edinburgh, Edinburgh, UK; b Shenzhen Zhong Nong Jing Yue Biotech Company Limited, Shenzhen, China; c College of Wildlife and Protected Area, Northeast Forestry University, Harbin, China; d Forest Inventory and Planning Institute of Jilin Province, Changchun, China

**Keywords:** *Anas zonorhyncha*, phylogenetic tree, mitochondrial genome

## Abstract

The complete mitochondrial genome of *Anas zonorhyncha* was first reported. The length of the entire mitochondrial genome was 16,605 base pairs, including 13 protein-coding genes, 22 tRNA genes, two rRNA genes, and a D-loop region. A phylogenetic tree of *A. zonorhyncha* was constructed with a group of related species in the family of Anatidae, indicating a close genetic relationship between *A. zonorhyncha* and *A. poecilorhyncha*.

Duck is poultry that has had close interactions with humans since ancient times. *A. zonorhyncha*, commonly known as Eastern spot-billed duck, was one of the ancestors of nowadays domestic ducks (Li et al. 2010). *A. zonorhyncha* was is mainly distributed in eastern Asia, and its breeding range stretched over a wide range of longitude, from Laos and Vietnam in the South to eastern Russia in the north (Wang et al. 2019). Increasing inbreeding between *A. zonorhyncha* and other closely related species within genus Anas was observed, and surprisingly, offspring was viable and fertile (Lavretsky et al. 2014). Due to the high resemblance of the genetic information between *A. zonorhyncha* and *A. poecilorhyncha* (mallard) (Wang et al. 2019), the taxonomy of these two ducks was in contention. Johnsgard and Mayr have placed *A. zonorhyncha* as a subspecies of *A. poecilorhyncha* (Johnsgard and Mayr 1979), while some other studies supported that they were sister groups under genus Anas (Johnsgard and Mayr 1979; Lavretsky et al. 2014). In this study, we generated the whole mitochondrial genome of *A. zonorhyncha* and provided insights into the phylogeny of *A. zonorhyncha*.

The muscle sample from an *A. zonorhyncha* individual collected at Changchun Beihu National Wetland Park (latitude: 125.3674 and longitude: 43.9678) was used for DNA isolation. This specimen was stored at Forest Inventory and Planning Institute of Jilin Province (http://lyt.jl.gov.cn/zsdw/jlslydcghy/, Huabing Guo, huabingguo9513@sina.com) with the voucher number LMX20180507. The research ethics is approved by the institutional ethical review board of Northeast Forestry University with the number 20210008. The genomic DNA was extracted from the muscle samples, and then fragmented for DNA library preparation and whole genome sequencing (WGS). About 8 Gb raw data were finally generated for performing a *de novo* assembly of the complete mitochondrial genome of *A. zonorhyncha* with NOVOPlasty (Dierckxsens et al. 2017). The mitogenome was annotated by MITOS2 (http://mitos2.bioinf.uni-leipzig.de/index.py).

The total length of the *A. zonorhyncha* mitochondrial genome was 16,605 bp, containing 13 protein-coding genes, 22 tRNA genes, two rRNA genes, and one control region (D-loop). Most of the genes were located on the heavy chain, except for ND6 and eight tRNA genes (tRNA^pro^, tRNA^glu^, tRNA^gln^, tRNA^ala^, tRNA^asn^, tRNA^cys^, tRNA^try^, tRNA^ser(TGA)^). tRNAs had relatively shorter gene lengths, ranging from 66 to 74 bp. Protein-coding genes were the dominant content, covering 68.7% of the entire DNA sequence. ND5 was the greatest gene within the genome, composed of 1824 base pairs. The majority of protein-coding genes used ATG as their start codon, while TAA was the most frequently used stop codon. *A. zonorhyncha* mitochondrial genome was made up of 29.2% of adenine (A), 22.2% of thymine (T), 32.8% of cytosine (C), and 15.8% of guanine (G). The composition did not show any obvious bias toward AT (51.4%) or CG content (48.6%), which was similar to closely related species like *A. platyrhynchos* and *A. poecilorhyncha* (Liu et al. 2019; Sun et al. 2020).

To reveal the evolutionary relationship between *A. zonorhyncha* and other teals, 13 protein-coding gene sequences were extracted from our assembled mitochondrial genome for constructing the maximum-likelihood tree by IQtree (Nguyen et al. 2015) with other 14 closely related species. The phylogenetic tree illustrated that *A. zonorhyncha* and *A. poecilorhyncha* were closely clustered together (Figure 1). The segregation between them might still be blurred, which might be a reason for fertile interbreeding offspring. If *A. zonorhyncha* and *A. poecilorhyncha* are evolve toward different pathways, more time may require for them to be further differentiated.

**Figure 1.**
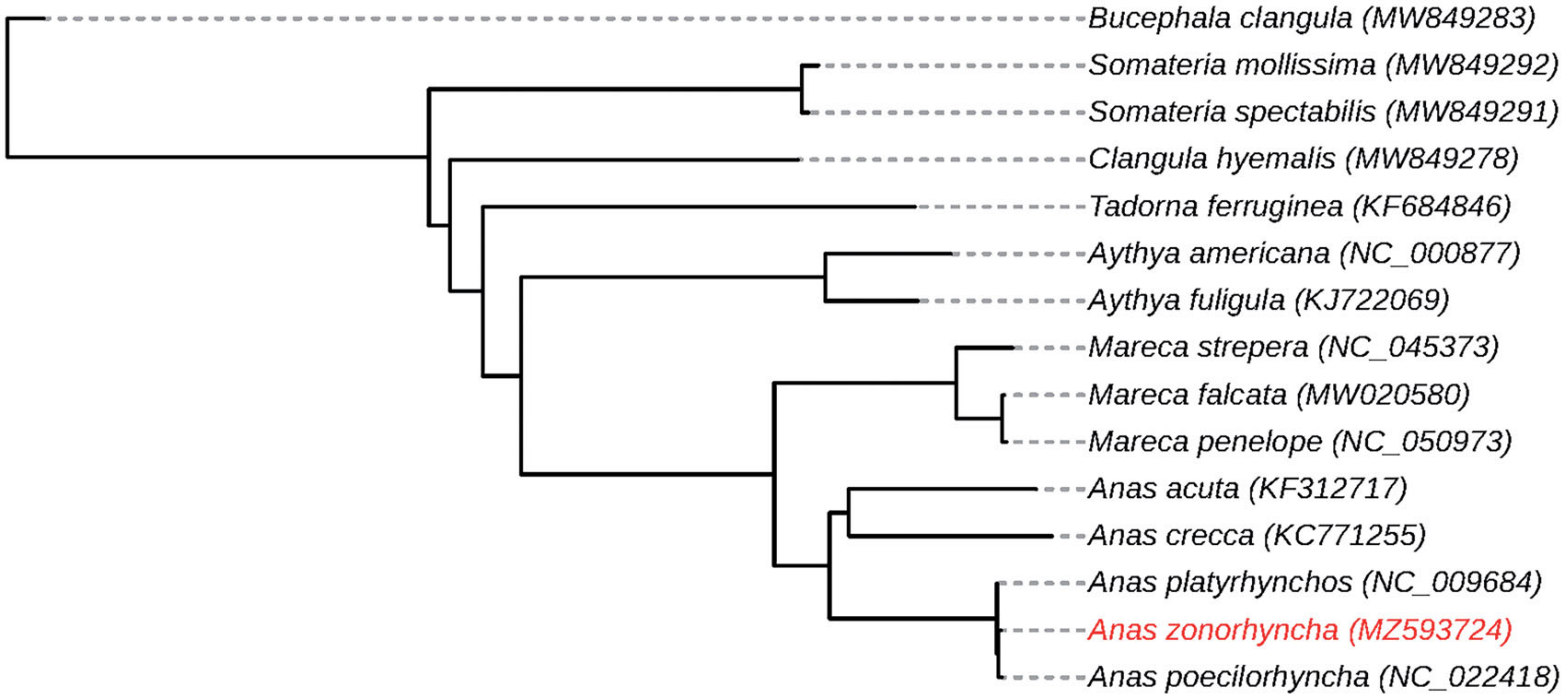
Maximum-likelihood phylogenetic tree of *A. zonorhyncha* with other species. The position of *A. zonorhyncha* was highlighted.

## Authors contributions

Huabing Guo and Kuo Xu were involved in the conception and design of the project. Kuo Xu and Chuyu Lin performed data analysis and computations. Kuo Xu drafted the paper. Xinyuan Zhang critically revised it for intellectual content. Huabing Guo gave the final approval of the version to be published. All authors agree to be accountable for all aspects of the work.

## Data Availability

The complete mitochondrial genome sequences generated in this study are openly available on NCBI website (http://www.ncbi.nlm.nih.gov) under the accession number MZ593724. The associated BioProject, SRA, and Bio-Sample numbers are PRJNA781902, SRR16991231, and SAMN23302468, respectively.
